# Strengthening of Structural Flexural Glued Laminated Beams of Ashlar with Cords and Carbon Laminates

**DOI:** 10.3390/ma15238303

**Published:** 2022-11-23

**Authors:** Agnieszka Wdowiak-Postulak

**Affiliations:** Faculty of Civil Engineering and Architecture, Kielce University of Technology, 25-314 Kielce, Poland; awdowiak@tu.kielce.pl; Tel.: +48-41-34-24-480

**Keywords:** glued laminated beams, fibre-reinforced timber, load-bearing capacity, stiffness, ductility, modulus of elasticity, bending test, carbon cords, carbon laminates, knots

## Abstract

Changes in the condition of existing timber structures can be caused by fatigue or biological attack, among other things. Replacing damaged timber is still very expensive, so it seems more advisable to repair or reinforce damaged elements. Therefore, in order to improve the static performance analysis of timber structures, reinforcement applications in timber elements are necessary. In this experimental study, technical-scale glulam beams measuring 82 × 162 × 3650 mm, which were reinforced with carbon strands and carbon laminates, were tested in flexure. A four-point bending test was used to determine the effectiveness of the reinforcement used in the timber beams. Internal strengthening (namely, glued carbon cords placed into cut grooves in the last and penultimate lamella) and an external surface of near-surface mounted (NSM) carbon laminates glued to the bottom surface of the beam were used to reinforce the laminated ashlar beams. As a result of this study, it was found that the bending-based mechanical properties of ash wood beams reinforced with carbon fibre-reinforced polymer composites were better than those of the reference beams. In this work, the beams were analysed in terms of the reinforcement variables used and the results were compared with those for the beams tested without reinforcement. This work proves the good behaviour of carbon fibre reinforced plastic (CFRP—Carbon fibre reinforced polymer) cords when applied to timber beams and carbon laminates. This study illustrated the different reinforcement mechanisms and showed their structural properties. Compared to the reference samples, it was found that reinforcement with carbon strings or carbon laminates increased the load-bearing capacity, flexural strength and modulus of elasticity, and reduced the amount of displacement of the timber materials, which is an excellent alternative to the use of ashlar and, above all, inferior grade materials due to the current shortage of choice grade. Experimental results showed that, with the use of carbon fibre (carbon cords SikaWrap^®^ FX-50 C—Sika Poland Sp. z o.o., Warsaw), the load bearing capacity increased by 35.58%, or with carbon cords SikaWrap^®^ FX-50 C and carbon laminates S&P C-Laminate type HM 50/1.4 - S&P Poland Sp. z o.o., Malbork, by 45.42%, compared to the unreinforced beams.

## 1. Introduction

Wood is anisotropic, hygroscopic and organic [1,2,3,4]. It should be remembered, also, that wood has many beneficial properties [5,6]. It represents a material that is advantageous for use in structures and in non-structural areas [7]. There is currently a rapid increase in the use of wood materials, primarily for structural applications. However, there are also certain limitations that allow the use of wood in the structural area. These limitations are: fibre twists, knots, cracks, difficulties in finding materials with the desired shapes and sizes, changes in production methods, production methods, use of materials with low strength properties in production, a high waste rate when using solid wood material, and the joining of short pieces. Therefore, wood as a material can be strengthened by supporting it with fibre-reinforced polymers both to increase the resistance of the strength properties of the joints of the structures to be obtained by using the wood material, and to repair damage caused by external factors such as material deterioration of previously built structures or due to earthquakes [1,8,9].

Wood construction makes an important contribution to the global energy consumption of greenhouse gas emissions. This has a significant impact on renewable materials, primarily timber structures. Although wood is a natural composite and is one of the oldest materials used in construction, it is important to remember that the use of wood and wood-based materials for structural purposes is still used today [4]. The use of timber as a structural element is the oldest known technique, especially in structural engineering projects, as an example of high deadweight construction. This is because these materials have a high strength-to-weight ratio and can be considered as highly sustainable materials. Recently, there has been a growing fondness for the use of timber in construction projects. This is triggered by its ability to hold dynamic loads and its mechanical properties, hence the many research works [10,11,12,13,14,15]. Currently, there are various structural timber products, and glulam is one of these products, described as one of the most efficient composite building materials. These glulam components consist of different layers of laminated timber, which are bonded together using a high-strength adhesive material to create a uniform component. Moreover, this causes a decrease in natural heterogeneities, such as knots occurring in the wood material [11,12,13,14,15,16,17,18,19]. Glulam elements make it possible to obtain elements with different dimensions and avoid inconsistencies in their properties. In recent years, a number of researchers have looked into the scope of glulam beams, so various experimental studies have been carried out. In a study by Anshari et al. [20], compressed timber layers in pre-cut rectangular holes were used to reinforce glulam beams. The study confirmed that the use of pressed timber layers as a reinforcing material is economically and environmentally effective. Another study [21] presented an investigation of the feasibility of glulam beams, the purpose of which was to determine the effective gluing factor, stating in its conclusions that the gluing parameters must be adjusted depending on the timber species. Another study [22] presented an investigation of the mechanical properties of the glue in a rod embedded in a glulam beam, where the results of an experimental pull-out test of this rod showed that failure occurred as a result of the rod slipping into the glulam beam and the delamination of the shear bond. Subsequently, the paper [23] demonstrated and concluded that reinforcement played an important role in the change of the failure form from brittle to ductile, which represented an increase in the load carrying capacity of the reinforced beams. Subsequent experimental studies [24] investigated bonded beams and carbon composites as reinforcing materials. Afterwards, it was found that the type and position of the reinforcement had a direct influence on the mechanical properties of the whole element. Subsequently, in [25], the influence of the toe joint profile of glued beams made of hardwood and its behaviour on the tensile strength was determined. As a result, it was suggested that the species studied could be used to produce glulam with a high tensile strength.

In recent years, the demand for wood has increased significantly, while timber is now a popular building material due to its ability to be used as a lightweight construction material. Ease of production or its unique physical and mechanical properties and low density are also enhanced by its attractive appearance. Due to its environmental protection or low energy requirements, wood is a commonly used construction material. Reinforcing or joining of wooden materials is usually done with steel elements. It is important to remember, however, that steel elements detach from the wood material over time and corrode. This decreases the quality of the environment over time, damages human health, shortens the life of the wooden element and also creates environmental problems. Therefore, nowadays most historic and existing wooden structures need to be safely repaired or reinforced. Known repair work, as well as insect infestation in various parts of the wooden elements over time, as well as fungal activity or decay, etc., can also cause environmental problems. On the other hand, leaving such elements that should be replaced for various reasons can cause serious problems both in terms of cost and structural safety. It would therefore be more appropriate to replace the damaged element rather than replace all the elements used in the building. Changes can be applied by using a dowel, a nail, or a blotting technique. However, this may not have the desired static result. On the other hand, reinforcing timber structures with FRP does not take a lot of time and at the same time provides an advantage also aesthetically [6]. Therefore, it is advisable to use man-made or natural fibres, whether based on basalt, glass, carbon, aramid, jute, etc., used in the study to reinforce wood [26,27,28,29,30,31,32,33,34,35,36,37,38,39]. It should be noted that performance reinforcing polymer (FRP) composites support the use, the care and reinforcement, of damaged structures. Their characteristics include their low height, high strength, high resistance to action and convenience and installation. Types of FRP include carbon fibre reinforced polymer (CFRP), glass fibre reinforced polymer (GFRP) and basalt fibre reinforced polymer (BFRP) composites. Due to the use of pultrusion technology, CFRP sheets can be rapidly developed with high forming properties. On the basis of experimental studies, the use of CFRP duct instead of externally bonded CFRP board was found to be a very effective way to renew or reinforce structures. This has the effect that the build-up rate of the CFRP plate can help 20–30% when the source is used. In addition, by using a specific compressed CFRP plate, load and shear capacity, deflection performance and crack growth can be effectively achieved. In addition, when providing prestressed CFRP for structural reinforcement, the end result creates the effect of the properties of the CFRP exposed to the generating temperature, water absorption and attachment with the necessity of loading. Although some analysis has been carried out to investigate the performance of FRP to study the environment, it should be noted that it is difficult to obtain the effect of the pressure level on the degradation of mechanical properties [40]. The paper [41] presents the results of a four-point bending test of fifteen small-size glulam specimens reinforced with glass (GFRP) or carbon (CFRP) cords, differing in the type of adhesive (epoxy resin or melamine glue). It was found that the effectiveness of the proposed technique was compromised by inadequate soaking of the installed cords and by too much resin. The reinforcements used were able to induce compressive failure of the upper timber layer once the beam cracked. In contrast, specimens reinforced with basalt cords showed better performance in terms of both strength and ductility. The average increase in strength was about 25%, while the average increase in ductility was about 40%, relative to unreinforced specimens. In an experimental paper [42], a programme for strengthening cross-laminated timber beams using carbon fibre reinforced polymer (CFRP) and glass fibre reinforced polymer (GFRP) composite sheets was presented. Encouraging results were found: the percentage increase in flexural stiffness was 26.29% and 45.76% for 2.5% and 5% addition of GFRP composite sheets on the tension side of the beam, respectively. However, for the same percentage addition of GFRP, the increase in flexural strength was 36.91% and 40% compared to the unreinforced beam. For a 1.67% and 3.33% addition of CFRP composite sheet to the tension side of the beam, the percentage increase in flexural stiffness compared to the unreinforced beam was 36.19% and 64.12%. Furthermore, the increase in flexural strength for the respective percentage additions of CFRP was 45.86% and 50.62%.

Due to its orthotropic natural properties, it must be taken into account that wood is a complex material and, therefore, analytical methods to describe its basic workings are limited. The presence of knots or cracks or skewness of the fibres also has a significant impact on the mechanical behaviour of a wood component, especially in the tensile zone that is present. For this reason, the material properties of wood can vary even between the same wood species, so many parameters are important and necessary to fully describe its modelling.

Based on an analysis of the literature, there has been some minor research carried out on carbon cord-reinforced laminated beams. In contrast, there has been virtually no research involving reinforcement with inferior carbon ropes or laminates with a reinforcement scheme such as that in the work below. Therefore, this paper presents the results of an experimental study carried out on glulam beams of medium and inferior quality class, with the aim of determining the effectiveness of an innovative strengthening technique involving the use of CFRP cords, instead of rods and CFRP laminates. Based on these considerations, further experimental studies are needed, for example by considering circular grooves.

## 2. Materials and Methods

### 2.1. Materials

Ash was the hardwood species that was chosen for the flexural testing of beams reinforced with CFRP materials. Obviously, the properties of this species are similar even to those of pine, primarily used in the construction industry, primarily in the manufacture of glulam or CLT (Cross Laminated Timber). It is a fast-growing, leaf-shedding and medium-sized tree, around 25–35 m in height and up to around 100 cm in diameter. In contrast to the trunks of trees growing in forests, its compactness is well-grown, long, straight, up to a height of about 20 m, and free of branches. In its slow-growing state, the tree is prone to bifurcation. The bark, up to about 50 years of age, is smooth and greenish-grey, then brown, almost black, and cracked. The proportion of bark is about 9–14% of the trunk volume with bark. Ash is found throughout Europe, from southern Scandinavia to the northern Mediterranean coastline (but not on the Apennine or Peloponnese peninsulas), and in Spain only in the north. In Poland, it is a wild forest tree ranked among the most valuable, noble species of native deciduous trees. It also requires fertile, deep, plump, moist or wet soils. It is very sensitive to frost. It does not form solid stands. It is found in river valleys, where it forms stands together with alder and oak.

The lamellas came from timber elements with strength classes D18—(f_m,k_—18 MPa, f_t,0,k_ —11 MPa, f_c,0,k_ —18 MPa, E_0,mean_ —9500 MPa, G_mean_–590 MPa) oraz D24—(f_m,k_ —24 MPa, f_t,0,k_ —14 MPa, f_c,0,k_ —21 MPa, E_0,mean_ —10,500 MPa, G_mean_—620 MPa), [43]. Smooth sawn timber beams, with a density of approximately 670 kg/m3, were reinforced with carbon fibre SikaWrap^®^ FX-50 C-carbon fibre cord, and SikaWrap^®^ FX-50 C and S&P C-Laminate type HM 50/1.4-carbon laminate.

According to the manufacturer, SikaWrap^®^ FX-50 C—Sika Poland Sp. z o.o., Warsaw [44] is a rope of unidirectionally aligned carbon fibres, encased in a foil sleeve, used as a surface mounted reinforcement, and provides a connector for anchoring SikaWrap^®^ mats. Unidirectionally aligned carbon fibres in a SikaWrap^®^ FX-50 C foil sleeve sheath with a dry fibre tensile strength of 4 GPa, 240 GPa dry fibre tensile modulus and a dry fibre density of 1.82 g/cm^3^. Sikadur^®^-330—Sika Poland Sp. z o.o., Warsaw [45] epoxy resin was used to bond the carbon fibre cords in the bonded beams in layers. The hardener and resin were mixed at a ratio of 1 to 4 by weight. Sikadur^®^-330 has the following parameters: flexural modulus E~3 800 MPa, tensile strength ~30 MPa, tensile modulus ~4500 MPa.

According to the manufacturer, S&P C-Laminates—S&P Poland Sp. z o.o., Malbork [46] are finished composite products made from carbon fibres embedded in an epoxy resin matrix. They are designed for the reinforcement of steel, reinforced concrete masonry and timber structures. Technical data are as follows: tensile strength ≥2800 N/mm^2^, modulus of elasticity ≥205 kN/mm^2^ and density 1.6 g/cm^3^. S&P Resin 55 HP—S&P Poland Sp. z o.o., Malbork [47] liquid epoxy resin was used to bond the carbon fibre laminates in the sandwich bonded beams to achieve a good wood—FRP bond. From the experimental studies already carried out, it was found that the best performance was obtained for the liquid epoxy resin, which penetrates very well into the pores of the wood; see [26]. S&P Resin 55 HP is a two-component, solvent-free adhesive based on epoxy resin with an amine hardener. The manufacturer’s technical data are as follows: modulus of elasticity ≥3200 N/mm^2^, compressive strength ≥ 100 N/mm^2^ and mixing ratio of components A:B is 4.2:1.8.

The wood species used in the study was ash, which has recently been increasingly used in the production of wood composites, and above all for structural purposes. For fibre composites, on the other hand, if high tensile strength is desired, then carbon fibres are an excellent material. Carbon fibre-reinforced polymers are the most common reinforcement applications used in industry. Other types of commonly used fibres are already significantly inferior to carbon fibres in terms of their modulus of elasticity or tensile strength, which makes them a suitable choice for reinforcing wooden beams in particular. In addition, carbon fibres have a high fatigue resistance, so that they can withstand significant stresses (for a certain number of cycles) without breaking. In addition, they are also non-combustible and have a low coefficient of thermal expansion. Although natural fibres appear to be a better choice in terms of their sustainability, natural fibres have a significantly lower tensile strength compared to carbon fibres. In contrast, natural fibres reduce greenhouse gas emissions. In this research, carbon fibre cords were used instead of natural fibres due to the potential increase in the mechanical properties of the composite. In contrast, the impact on greenhouse gas creation potential could be offset if recycled carbon fibres were used instead of virgin carbon fibres.

### 2.2. Samples Preparation

The timber samples were selected from pieces with natural defects, such as knots, to investigate the possibility of which fibres could address the natural defects in the timber. The timber samples were cut from the same trunk (with the timber fibres parallel to the length of the beam) to reduce differences that could affect the comparison of results. All the timber elements from which the glued laminated beams were made were visually sorted by strength methods, dividing them into sorting classes: KS—medium quality class, and KG—lower quality class, according to PN-D-94021:2013-10 [48]. The experimental tests mainly focused on the analysis of the increase in load-bearing capacity compared to the load-bearing capacity of unreinforced elements.

The timber beams were grooved in designed square patterns for the placement of the reinforcement, so as to provide an opportunity to lay the carbon fibres as cords on the timber beams, The carbon fibres were laminated onto the beams using an epoxy resin mix and allowed to dry for 24 h.

Considering the type of reinforcement material, 15 unreinforced and reinforced laminated timber beams were designed and designated as NCFRP (5 units) as unreinforced beams, followed by CFRP1 (5 units) as reinforced with carbon strands with a degree of strengthening of 2.51% and CFRP2 (5 units) as reinforced with both carbon strands and carbon laminates with a degree of strengthening of 3.07%.

Four grooves were made along the length of the timber beams; next, the cords were placed in the cut-out, then these were bonded to the timber using epoxy resin. This reinforcement technique would allow significant bending strength to be achieved in the designed beams while maintaining shallow depths. It would therefore allow for a long-span structure with reduced dimensions and a significant visual effect.

**Figure 1:**
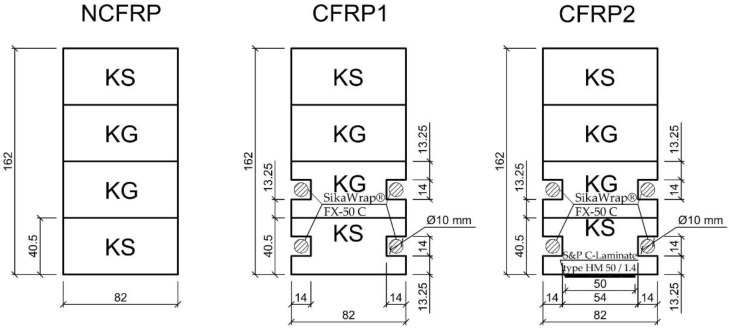
Cross-section of the laminated beams.

All the beams were made with the same external section size: their length was 3650 mm, the span between supports was 3000 mm, the height was 162 mm and the width was 82 mm. The beams, designated CFRP1, were reinforced with carbon fibre cords of approximately 10 mm in diameter and 3750 mm in length. The CFRPs were bonded in holes of approximately 14 mm to the compressed carbon fibres using Sikadur^®^-330 epoxy adhesive. The square holes around the carbon cords were filled with this epoxy adhesive. S&P Resin 55 HP epoxy adhesive, on the other hand, was applied to the primed surfaces using a roller to a thickness of 1 mm. Substrate preparation was carried out by planing the beam elements. The resulting dust was removed with a hoover. The surface was primed using S&P Resin 55 HP system adhesive immediately before bonding with S&P C-Laminate. A diagram of the reinforcement including dimensions is shown in Figure 1. The prepared beams were stored for 1 week before bending tests. Prior to testing the beams, all elements were stored at a temperature of 20 ± 2 °C and a relative humidity of 65 ± 5%. The moisture level was checked with an electric moisture meter at 12%.

### 2.3. Test Methods

**Figure 2:**
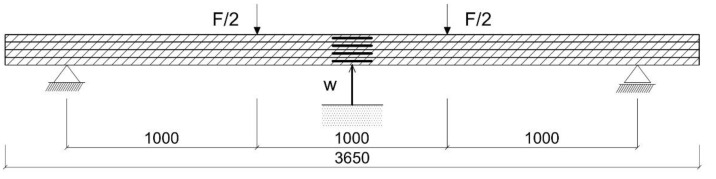
Four-point bending test.

The bending tests were carried out on timber beams according to EN 408+A1:2012 [49] and EN 1995-1-1:2010 [50]. All the beams were tested at a span of 3000 mm in four-point bending. Steel plates, 50 mm wide and 15 mm thick, were placed at the bearing and support points to relieve displacement rotation and to recreate a simply supported beam. A freely supported glulam beam is shown in Figure 2. The four-point bending experimental test was carried out using a mechanical testing machine. In order to delineate the change in strain at the mid-span of the specimen during the entire loading process, the measuring points were evenly distributed along the height. The deformations were tested using a ‘Demec’ extensometer. The span of the support points was taken as 203.2 mm. The deflection tests, on the other hand, were determined at the centre of the beam span using dial gauges. The four-point bending test as a representation of the experimental setup is shown in Figure 2.

## 3. Results

In this study, laminated beams were reinforced with carbon fibre: SikaWrap^®^ FX-50 C or S&P C-Laminate type HM 50/1.4 carbon fibre CFRP. The reference and reinforced beams were subjected to bending tests.

### 3.1. Load-Displacement Responses

**Figure 3:**
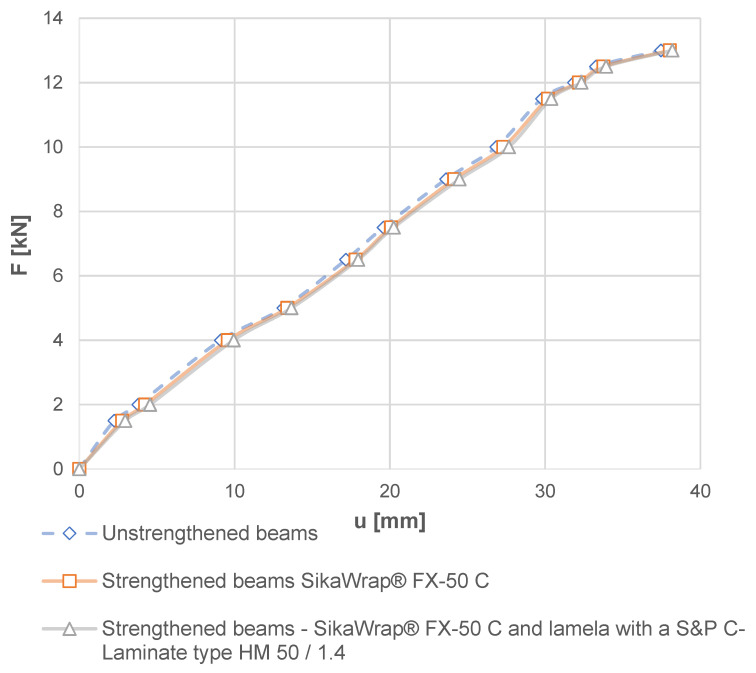
Load-displacement diagrams of FRP-strengthened and unstrengthened beams.

**Figure 4:**
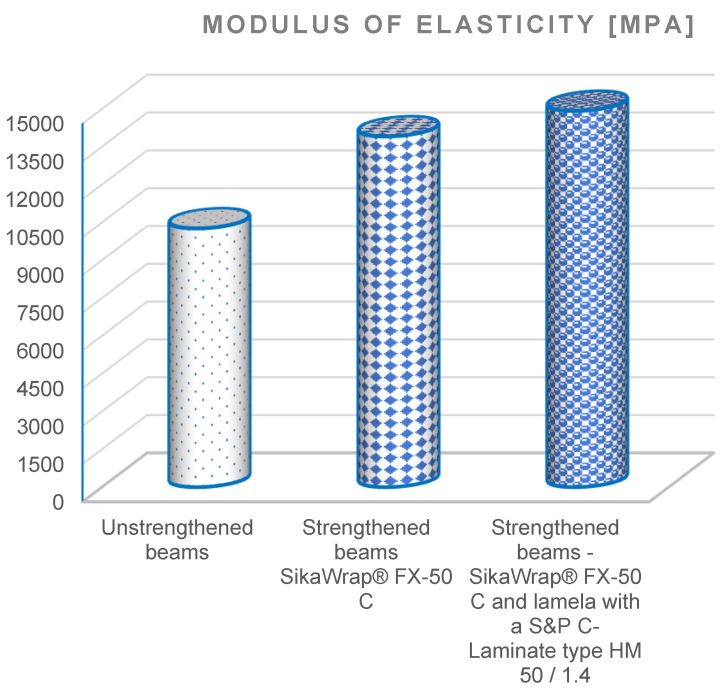
Diagrams of the moduli of elasticity of strengthened and unstrengthened beams.

**Figure 5:**
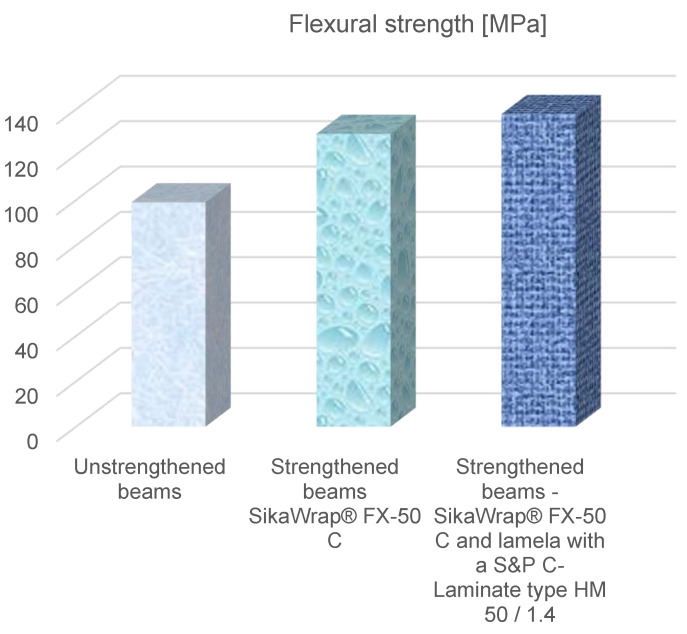
Bending strength diagrams of the strengthened and reference beams.

Load-displacement diagrams and the bending strength and modulus of elasticity values obtained in the test are given in Figure 3, Figure 4 and Figure 5.

From the tests, it was found that the load capacity of the elements reinforced with carbon fibre SikaWrap^®^ FX-50 C increased by 35.58% or with SikaWrap^®^ FX-50 C and S&P C-Laminate type HM 50/1.4 carbon fibre CFRP by 45.42% with the reference beams. It was found that the displacement volume with the SikaWrap^®^ FX-50 C reinforced beam increased by 15.22% and with the SikaWrap^®^ FX-50 C reinforced beam and S&P C-Laminate type HM 50/1.4 CFK carbon fibre CFRP laminates by 19.49%, compared to the reference beams.

After analysing Figure 4 and Figure 5, it was found that beams reinforced with SikaWrap^®^ FX-50 C carbon fibre–carbon ropes, had a flexural strength (129.22 MPa) and modulus of elasticity (13,910 MPa), while beams reinforced with SikaWrap^®^ FX-50 C and S&P C-Laminate type HM 50/1.4 had a flexural strength (137.97 MPa) and modulus of elasticity (14,920 MPa). It was found that the flexural strength value of the reinforced SikaWrap^®^ FX-50 C beam increased by 31.53%, SikaWrap^®^ FX-50 C and S&P C-Laminate type HM 50/1.4 by 39.36%, whereas the modulus of elasticity of the SikaWrap^®^ FX-50 C increased by 35.58%, and SikaWrap^®^ FX-50 C I laminate S&P C-Laminate type HM 50/1.4 by 45.42%.

### 3.2. Deformations of Unstrengthened and Strengthened Beams

**Figure 6:**
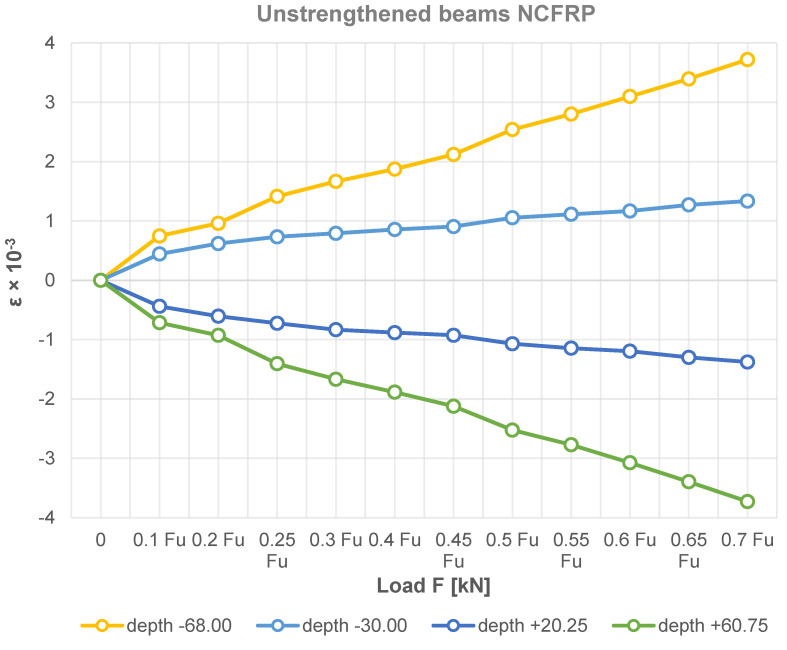
The distribution of deformations in timber beams at the section height.

**Figure 7:**
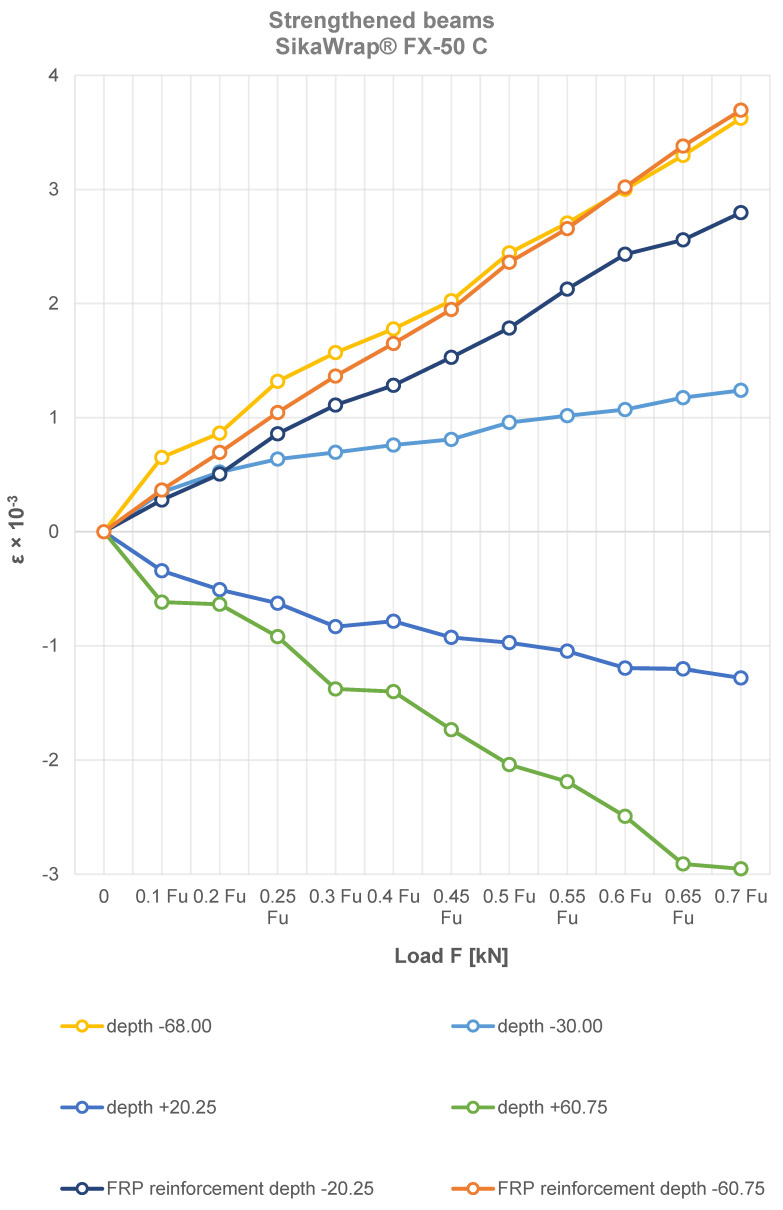
The distribution of deformation in timber beams and FRP materials at the section height.

**Figure 8:**
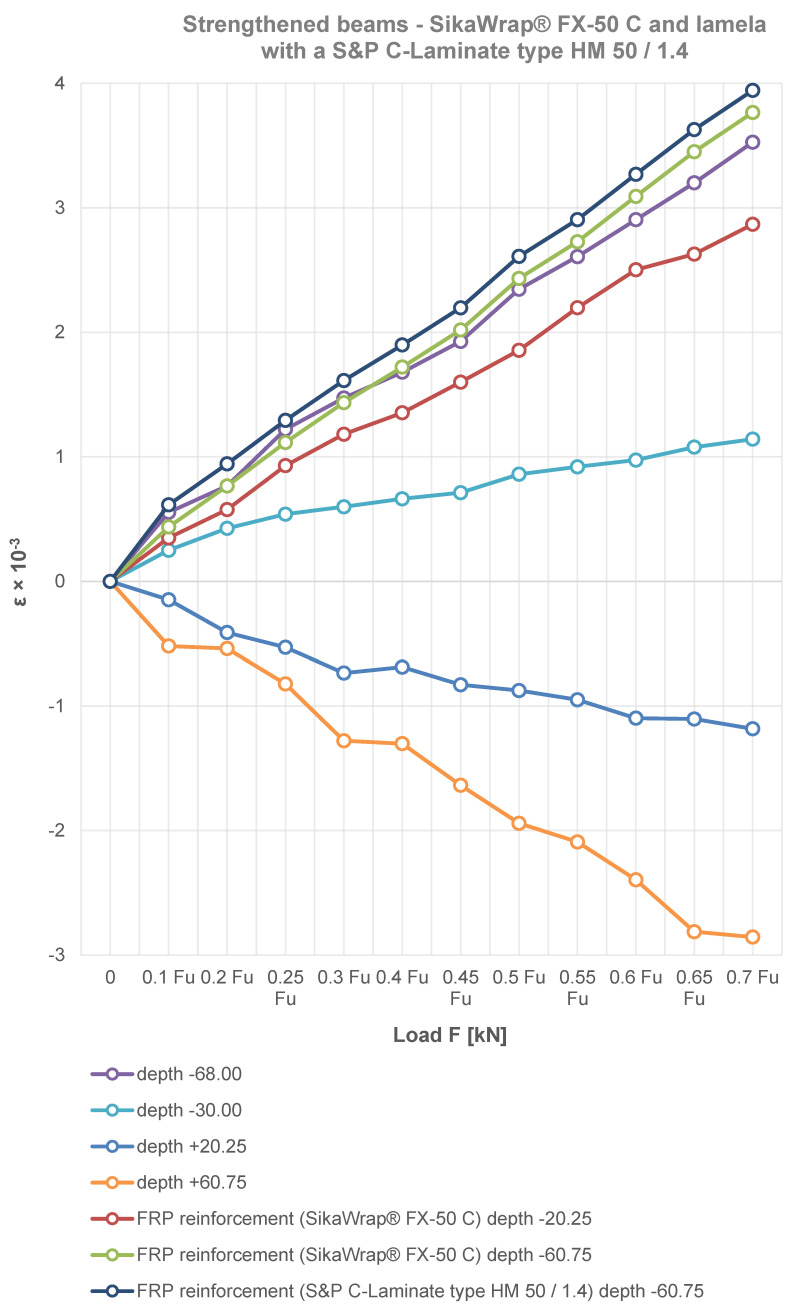
The distribution of deformation in timber beams and FRP materials at the section height.

The variation of the bending strain of the reinforced beams at the height of the section in the middle span and the FRP materials used are shown in Figure 6, Figure 7 and Figure 8. The figures show that the compressive strain is negative, while the tensile strain is positive. In contrast, the strain at the height of the glued beam varies linearly, which is obviously consistent with the assumption of a planar section. Note that the neutral axis in some cases of SikaWrap^®^ FX-50 C or S&P C-Laminate type HM 50/1.4 reinforced beams shifts downwards. In the experimental process of loading laminated timber beams, the timber in the compression zone is in a plastic state, while it can be seen that the applied reinforcements, i.e., SikaWrap^®^ FX-50 C or S&P C-Laminate type HM 50/1.4 CFRP in the tension zone, have an increasingly significant effect on the static performance analysis of the beams.

### 3.3. Effect of CFRP Reinforcement on Beam Bending

As can be seen in the experimental results in Figure 3, Figure 4, Figure 5, Figure 6, Figure 7 and Figure 8, the elastic modulus of the FRP materials increased with the increasing percentage of reinforcement. The lower the elastic modulus, the smaller the deformation was. In the case of beam elements reinforced with carbon strings and carbon laminates, it proved to be the most effective, providing up to a 39.36% additional increase in load capacity compared to the unreinforced condition (see Figure 6, Figure 7 and Figure 8).

Based on the experimental tests carried out, due to the selective placement of the FRP material in the areas of the beam subjected to stronger tension, the wood fibres in the upper part of the beam produced plastic compressive deformation (see Figure 7 and Figure 8).

Figure 3, Figure 4, Figure 5, Figure 6, Figure 7 and Figure 8 clearly show a significant increase in the load carrying capacity of the carbon fibre reinforced beams compared to the reference beams. Furthermore, in Figure 3, it can be seen that the elongation of the beam improved significantly after strengthening. It can be seen from the experimental study that the more the beams were reinforced, the more they showed a plastic curve.

On the basis of the research, it was also found that beam elements can also be used for building structures as well as components that can also be subjected to sudden impact loads such as from earthquakes at the same time. It should further be noted that, on the basis of the bending test, it was noted in order to increase the ductility of the reinforced section, it is recommended that the timber elements do not have natural defects. Furthermore, it was noted that, by analysing the strain distribution, fibre reinforcement in the tensile zone of the member maximises the effective use of the material in the compressive zone. In the experimental tests, it was further noted that during the tests the laminated beams or reinforcing materials did not slip as well as creep.

For the group of beams reinforced with carbon cords or ropes and carbon laminates, the compressive strain was slightly lower than the tensile strain at the initial loading stage. The strain distribution in the four-point bending of reinforced beam elements was initially characterised by elastic deformation of the laminated beams, followed by an initial elastic-plastic deformation, then elastic-plastic deformation. Subsequently, the deformation reached the elastic final tensile deformation in the tensile zone, improved by carbon reinforcement.

From the beginning of the load up to about 70 per cent of the failure load, the object– load–displacement curve was linear (Figure 3), which at the same time shows linear elastic characteristics. Thereafter, until the final load point was reached, the curve is non-linear. Thus, it can be seen that the laminated beam behaved plastically to a certain extent as the load increased. Subsequently, the beam underwent failure, in which the stiffness of the laminated beam increased continuously with the increasing load to the effect of reaching total failure. In contrast, once reinforcement was applied in the tension zone, the ductility of the sandwich glued beam was then fully exploited and failure occurred in the compression zone. In some beams, the failure occurred from vertical cracks in the middle zone. In addition, the remaining beams experienced wood fibre cracking longitudinally in the shear zone between the support and the point of force application. The failure in most of the unreinforced beams was related to longitudinal cracking as delamination of the wood grain, most commonly in the wood defects, was present. Three beams failed with no cracks, and no separation of the CFRP laminate. The failure was associated with longitudinal cracking as destruction between the support and the load application point, usually at the top of the section. On some beams, the longitudinal crack appeared in the lower part of the section. That is, it was related to shear. There was usually no peeling of the CFRP laminate from the glue or the glue from the timber. On the other hand, the beam behaved differently when it was subjected to rapid damage related to the peeling off of the CFRP laminate. The destruction occurred from the tearing of part of the timber member from the lower edge of the beam near the support; this was associated with shear cracks and carbon cords.

In this study, no CFRP reinforcement failed. The yield strength of the CFRP is higher than the yield strength of wood. It was observed in the study that complete failure occurred when the mid-span deflection was over 60.16 mm. This value is considered a high value, which provides good results from the point of view of ductility, as a result of which people have enough time to escape from the building before collapse. The study also concluded that there was a significant increase in ductility. It was noted that, even after the final collapse, the beams were still united. Therefore, there was no catastrophic failure when the beams were reinforced with CFRP. What is important, as a ductile material in design, is that it is possible to make ductile timber beams by adequate reinforcement of the tension zone, where it is possible to design reinforced timber beams up to yield strength, as is the case with steel structures.

## 4. Conclusions

On the basis of experimental tests carried out on glued laminated timber beams made of ash reinforced with Sika Wrap^®^ FX-50 C carbon strings and S&P C-Laminate type HM 50/1.4 carbon laminates, it was concluded that:

(1) Research has shown that ashlar timber used for glulam beams has become an important and attractive alternative in today’s construction industry. It can be seen that wood is now being used to supplement or even replace concrete structures in composite and hybrid systems. On the basis of the study, it was also noted that the FRP reinforcement technology used (SikaWrap^®^ FX-50 C carbon cords or S&P C-Laminate type HM 50/1.4 carbon cords) for the reinforcement of ashlar timber elements is structurally very effective and can also complement concrete structures. It should be noted that wood beams reinforced with carbon fibre in the form of cords or laminates can be used both to repair existing structures and to build new ones. It should also be noted that carbon fibres as FRP materials also have a high deformation tolerance and a low coefficient of thermal expansion.
(2) In a four-point bending experimental study, the effect of carbon reinforcement on the properties of the wood material of laminated beams made of ash graded KS and KG was investigated. Compared to reference samples, it was found that reinforcement with carbon strings or carbon laminates increased the load capacity, bending strength and modulus of elasticity, and the magnitude of displacements of the timber materials, which is an excellent alternative to the use of ash and, above all, inferior grade materials due to the current shortage of choice grade.
(3) With reference to the experimental studies carried out regarding the four-point test of the effectiveness of the applied reinforcement in the form of cords or CFRP laminates in laminated reinforced beams, there was an average increase in the ultimate load of 30.51% and 39.36% for reinforcement ratios of 2.51% and 3.07%, respectively. With this performance of reinforced members, this allows for the use of lower height members, which can be an alternative for projects with architectural constraints.
(4) Based on the tests, it was found that laminated beams with an applied reinforcement ratio of 2.51% and 3.07% showed higher average displacements of 15.22% and 19.49%, respectively. In addition, it was noted that, at higher loads and higher displacements, the reinforced beams exhibited more ductile behaviour.

The results confirmed the analyses of experimental studies, which showed the effectiveness of the use of cords or carbon laminates in increasing the load-bearing capacity of beams, as well as ductility. In addition, load-displacement and strain diagrams are shown as part of the verification of the study, enabling a procedure for the dimensioning of components reinforced with cord and carbon laminates. However, further research is needed with different types of wood or reinforcement, as well as with different dimensions and loads.

## Data Availability

Not applicable.
